# Randomized Trial of Chronic Pain Self-Management Program in the Community or Clinic for Low-Income Primary Care Patients

**DOI:** 10.1007/s11606-017-4244-2

**Published:** 2018-01-03

**Authors:** Barbara J. Turner, Yuanyuan Liang, Maureen J. Simmonds, Natalia Rodriguez, Raudel Bobadilla, Zenong Yin

**Affiliations:** 1 Department of Medicine, Long School of Medicine, University of Texas Health San Antonio, San Antonio, TX USA; 2 Center for Research to Advance Community Health (ReACH), University of Texas Health San Antonio, San Antonio, TX USA; 3 Department of Epidemiology and Public Health, Division of Biostatistics and Bioinformatics, University of Maryland School of Medicine, Baltimore, MD USA; 4 Department of Physical Therapy, School of Health Professionals, University of Texas Health San Antonio, San Antonio, TX USA; 5 Department of Kinesiology, Health and Nutrition, University of Texas at San Antonio, San Antonio, TX USA

**Keywords:** chronic pain, self-management, low-income populations, Hispanic, patient engagement

## Abstract

**Background:**

Patients with chronic pain often lack the skills and resources necessary to manage this disease.

**Objective:**

To develop a chronic pain self-management program reflecting community stakeholders’ priorities and to compare functional outcomes from training in two settings.

**Design:**

A parallel-group randomized trial.

**Participants:**

Eligible subjects were 35–70 years of age, with chronic non-cancer pain treated with opioids for >2 months at two primary care and one HIV clinic serving low-income Hispanics.

**Interventions:**

In one study arm, the 6-month program was delivered in monthly one-on-one clinic meetings by a community health worker (CHW) trained as a chronic pain health educator, and in the second arm, content experts gave eight group lectures in a nearby library.

**Main Measures:**

Five times Sit-to-Stand test (5XSTS) assessed at baseline and 3 and 6 months. Other reported physical and cognitive measures include the 6-Min Walk (6 MW), Borg Perceived Effort Test (Borg Effort), 50-ft Speed Walk (50FtSW), SF-12 Physical Component Summary (SF-12 PCS), Patient-Specific Functional Scale (PSFS), and Symbol–Digit Modalities Test (SDMT). Intention-to-treat (ITT) analyses in mixed-effects models adjust for demographics, body mass index, maximum pain, study arm, and measurement time. Multiple imputation was used for sensitivity analyses.

**Key Results:**

Among 111 subjects, 53 were in the clinic arm and 58 in the community arm. In ITT analyses at 6 months, subjects in both arms performed the 5XSTS test faster (−4.9 s, *P* = 0.001) and improved scores on Borg Effort (−1, *P* = 0.02), PSFS (1.6, *P* < 0.001), and SDMT (5.9, *P* < 0.001). Only the clinic arm increased the 6 MW (172.4 ft, *P* = 0.02) and SF-12 PCS (6.2 points, *P* < 0.001). 50ftSW did not change (*P* = 0.15). Results were similar with multiple imputation. Five falls were possible adverse events.

**Conclusions:**

In low-income subjects with chronic pain, physical and cognitive function improved significantly after self-management training from expert lectures in the community and in-clinic meetings with a trained health educator.

**Electronic supplementary material:**

The online version of this article (10.1007/s11606-017-4244-2) contains supplementary material, which is available to authorized users.

## INTRODUCTION

Although experts endorse non-pharmacologic interventions as first-line treatment for chronic pain management,^1^ low-income patients often lack access to these approaches. A practical chronic pain self-management program integrating components of a functional restoration program^2^ may offer a valuable resource for vulnerable populations. Based on community-based participatory research principles,^3^^,^ 4 the Living Better Beyond Pain/Vivir Mejor Más Allá del Dolor self-management program was developed to address unmet needs prioritized by rural, predominantly Hispanic stakeholders with chronic pain.^5^ Examples of unmet needs included increased chronic pain support/counseling, education about pain, exercise, massage, and weight control.^5^ The community stakeholders also endorsed chronic pain support from professionals and other community members. To evaluate outcomes from the 6-month Living Better Beyond Pain program, we conducted a parallel-group 6-month trial, randomizing subjects to group lectures by content experts in a community setting or individual meetings in a clinic setting with a community health worker (CHW) trained as a chronic pain health educator. Similar to the community stakeholders who generated priorities for the training program, eligible subjects were low-income, mainly Hispanic patients with chronic pain.

The community stakeholders endorsed improved physical function as an important goal. Thus, the primary outcome measure was the five times sit-to-stand test (5XSTS) to objectively assess lower extremity strength and balance.^6^ Given the pleomorphic effects of chronic pain, secondary outcomes included pain severity and nine measures of physical, cognitive, and psychological function. This analysis reports results for the main outcome, the 5XSTS test, and all six secondary measures of physical and cognitive function. This trial was designed specifically to evaluate two relatively low-cost approaches for providing pain management education and support to patients in communities with limited access to resources.

## METHODS

### Setting and Sample

Study subjects were recruited from academic general internal medicine, family medicine, and HIV clinics that were affiliated with the University of Texas Health Science Center at San Antonio (UT Health San Antonio) and that treated low-income, primarily Hispanic patients. From the electronic medical record, we identified patients aged 35–70, English- or Spanish-speaking, with chronic low back or lower extremity pain, and who were prescribed opioid analgesics for >2 months within the past year. Exclusions included cancer pain, significant mental health disorder, alcohol or drug abuse, inability to walk unassisted one block, inability to provide informed consent (e.g., dementia), and living over 10 miles from the clinic. Eligible subjects received a letter from the clinic director and a recruitment call from the study coordinator (N.R.) or a CHW (R.B.). All participants provided written informed consent. The institutional review board of UT Health San Antonio approved this study and all components of the educational program (HSC20150600H).

### Chronic Pain Self-Management Program

The Living Better Beyond Pain/Vivir Mejor Más Allá del Dolor training program addressed community stakeholders’ priorities^5^ and themes from other self-management programs for high-literacy patients: Explain Pain,^7^ the Progressive Goal Attainment Program (PGAP),^8^ the Pain Toolkit,^9^ and the Community Health Association of Mountain/Plains States (CHAMPS).^10^ Eight Living Better Beyond Pain topics were presented on PowerPoint slides in English or Spanish at a sixth grade reading level (Online Appendix A). All subjects received a notebook with copies of slides for each topic and photos of local Hispanic community members performing stretching and strengthening exercises at different levels of difficulty. Additional materials included activity logs with personal goals, program DVDs (walking exercises, self-massage techniques), exercise mats, tennis balls for massage, and multi-pronged self-massage tools. All subjects were instructed not to attempt activities that were too difficult, such as floor exercises or walking without support.

A bilingual CHW (M.R.) received 10 hours of training to serve as a chronic pain health educator. The training was provided by content experts and included curriculum, motivational interviewing, and proper demonstration of stretching and exercising activities. Before the program was initiated, this individual practiced delivering the sessions with other CHWs and the research team members. The project leaders (B.T., M.S., Z.Y.) met with the health educator every 1–2 months to review upcoming content to ensure fidelity to the program.

### Randomization

Randomized assignment was performed using sealed opaque envelopes opened after baseline study measurements were obtained. In light of library and clinic space limitations, the same pain self-management program was offered in two waves: wave 1 was conducted from February 1 to August 1, 2016, and wave 2 from June 1 to December 30, 2016.

### Study Arms

In the community arm, nine 1 hour group meetings were held at a local library—every 2 weeks for 3 months, then monthly for 3 months; the same session was offered twice weekly. One of these meetings involved a presentation on the library’s health information resources and effective use of internet search engines. The curriculum was translated into Spanish and back-translated to compare for accuracy. Physical therapy students demonstrated exercises, and the group practiced with supervision.

For the clinic arm, the health educator held six monthly one-on-one meetings for 30–45 min. The eight core lectures were condensed into six, but the same PowerPoint slides were reviewed and exercises demonstrated. To facilitate attendance, the timing of meetings coincided with office visits whenever possible.

All study subjects selected personal goals for physical activities, aiming for 30 min of light to moderate exercise on most days, and with attention to safety. Additional goals included practicing mindfulness and dietary changes. Missed meetings could be rescheduled. Per protocol, subjects received at least one phone call between visits from the CHW (community) or the clinic health educator (clinic) to review progress, and were reminded about upcoming meetings by phone or text message. Baseline and follow-up measures were conducted by physical therapy students, CHWs, or team members who were not involved in that study arm.

### Primary Outcome Measure

Our primary outcome was the five Times-Sit-to-Stand test (5XSTS) measures for standing from a standard armless chair, averaged after two trials.^6^^,^ 11 ^,^ 12

### Secondary Outcome Measures

Physical function measures included the 6-Min Walk test (6 MW)^13^; *Borg Perceived Effort Test* (Borg Effort) after completion of the 6 MW^14^; and *50-ft Speed Walk test* (50FtSW)^11^; the *12-Item Short-Form Survey Physical Component Summary* (SF-12 PCS)^15^; and *Patient-Specific Functional Scale* (PSFS), which asks about limitations to performing specific activities, ranking each in importance, in order to track progress.^16^ A modified version of the SF-12(v1) was used that allowed for English and Spanish subject data to be combined. To assess cognitive function, the *Symbol–Digit Modalities Test* (SDMT) evaluates attention and psychomotor speed.^17^^,^ 18 Secondary measures not reported here include the SF-12 Mental Component Summary, Brief Pain Inventory, Patient Health Questionnaire-9, and the Tampa Scale for Kinesiophobia. All measures were performed at baseline and 6-month study endpoint, but the following measures were also assessed at 3 months: 5XSTS, 50FtSW, PSFS, PHQ-9, and SDMT.

### Other Covariates

Patient demographics included age at baseline, sex, race/ethnicity, primary language preference, marital status, and employment status. Other characteristics included insurance type, body mass index (BMI), maximum pain on an 11-point scale, and pain location.

### Analysis

This study evaluates outcomes of delivering chronic pain self-management training with group lectures in a community setting or individual meetings in a clinic setting. Because the main outcome was the 5XSTS test, power was based on our pilot study of local veterans with chronic pain on opioids who received peer support and improved on the 5XSTS by a mean 10 s (SD = 12.8).^19^ A sample of 35 patients in each study arm provided 99% power to detect this difference (*P* < 0.05) using a two-sided one-sample *t* test. We aimed for 55 subjects per group to account for dropout.

Differences by study arm were examined using the *t* test or Mann–Whitney *U* test for continuous variables and chi-square test or Fisher’s exact test for categorical variables. Comparison of baseline characteristics for subjects who dropped out after baseline (*n* = 42) with those completing follow-up measures (*n* = 69) was conducted using the Mann–Whitney *U* test for continuous measures and chi-square test for categorical measures.

To evaluate pre–post changes within each group, change from baseline to 6 months (or baseline to 3 months) was assessed using a one-sample *t* test and effect size (i.e., Cohen’s *d*) computed (small 0.2 to <0.50, medium 0.50 to <0.80, and large ≥0.80).^20^ The difference in change for each outcome was compared for the two study arms using the Mann–Whitney *U* test.

To evaluate outcomes from the trial following the intention-to-treat (ITT) principle, linear mixed-effects models (LMMs) were used to examine the intervention effect among all randomized subjects using all available measures (baseline, 3 months, and 6 months), adjusting for time, study arm, sex, ethnicity (or survey language), age, BMI, and baseline maximum pain. We examined interactions between time and study arm; these were removed from the final model if not statistically significant. Scheffé’s method was used to examine change in an outcome of interest (i.e., 3-month vs. baseline; 6-month vs. baseline), adjusting for multiple comparisons.

In a sensitivity analysis, a multiple imputation with chained equations (MICE) approach was used to impute missing data at any point in the study.^21^ Imputation adjusted for time of assessment, study arm, sex, ethnicity, age, BMI, and baseline maximum pain. Ten imputed data sets were created. LMMs were fitted for each imputed data set and effect sizes were combined following Rubin’s rules.^22^ All analyses were performed using Stata/SE (version 14.1, 2015; StataCorp LP, College Station, TX).

## RESULTS

**Figure 1:**
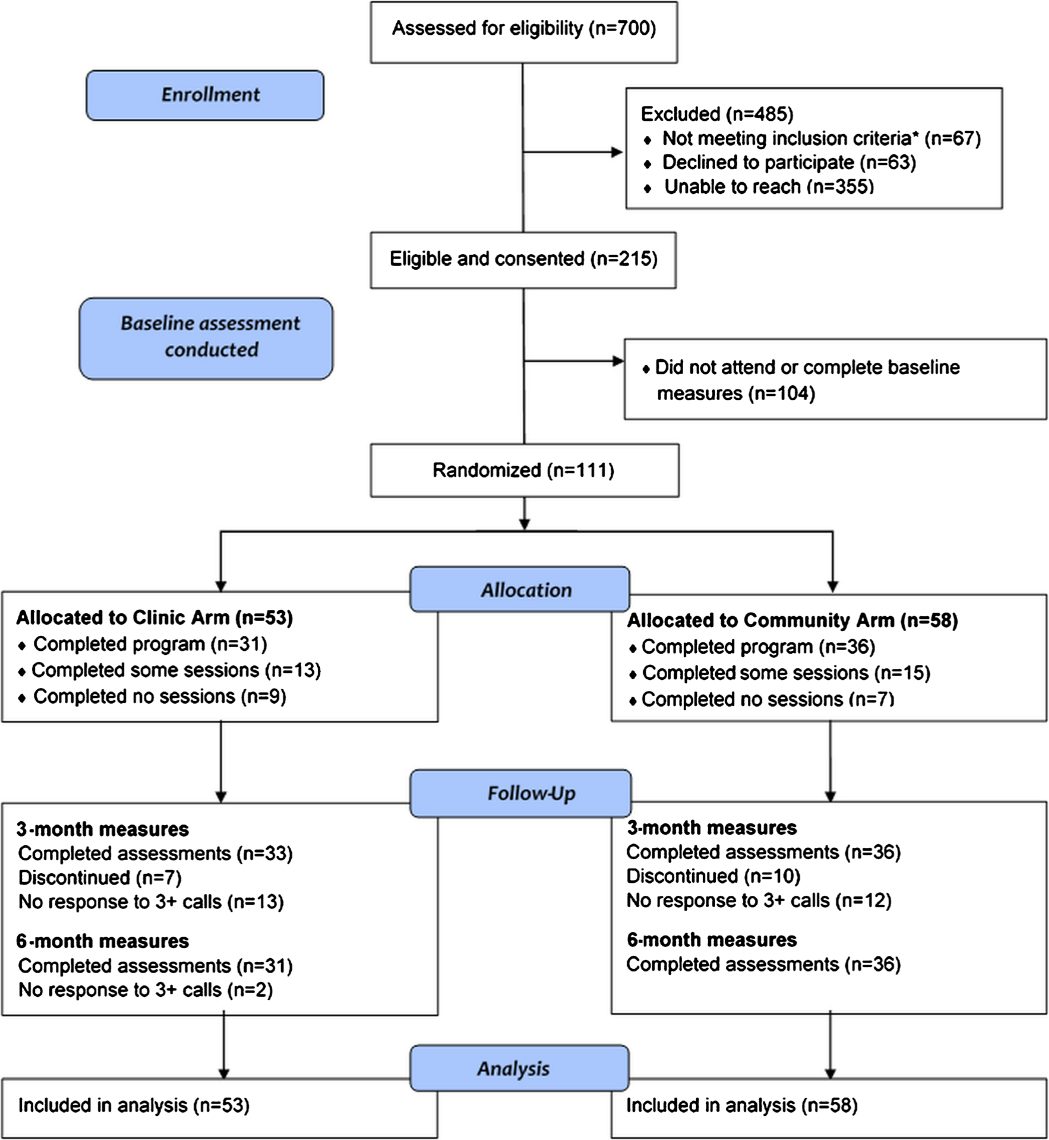
Consolidated Standards of Reporting Trials (CONSORT) flow diagram. *Medical exclusions: severe medical or psychiatric comorbidity, cancer pain, mild pain, unable to walk, pregnant, surgery.

Seven hundred eligible subjects were identified from the electronic medical record, but half could not be reached by phone (Fig. 1). Of 111 randomized subjects, 53 (47.7%) were assigned to the clinic arm and 58 (52.3%) to the community arm (Fig. 1). Among clinic arm subjects, 33 (62.3%) completed 3-month measures and 31 (58.5%) completed 6-month measures. Of the 31 who completed the program, 21 (71%) rescheduled at least one meeting, and one subject failed to make up two missed meetings. In the community arm, 36 (62.1%) subjects completed measures at both time points, and 23 (64%) missed at least one lecture meeting, which was received in separate group meetings with the CHW assisting the community arm or another lecture.

Among all subjects, the mean age was 56.5 years, over half were women, and over three-quarters were of Hispanic ethnicity (Table 1). About one-quarter preferred to communicate in Spanish, and 30% were married. Most subjects were not working, roughly one-third were enrolled in Medicare, and another third were uninsured. On average, study subjects had a mean BMI of 35, and most reported having pain in multiple locations.

**Table 1:** Baseline Characteristics of Study Subjects Randomized to Community Arm or Clinic Arm

Variables	Community Arm^a^ *N* (%)	Clinic Arm^a^ *N* (%)	All *N* (%)
Sociodemographic Subject characteristics	58 (52)	53 (48)	111 (100)
Age, mean ± SD (years)	56.9 ± 8.7	56.2 ± 9.4	56.5 ± 9.0
Women	37 (63.8)	24 (45.3)	61 (54.9)
Race/ethnicity			
Hispanic	45 (77.6)	42 (79.2)	87 (78.4)
Non-Hispanic white	8 (13.8)	6 (11.3)	14 (12.6)
Non-Hispanic black	5 (8.6)	5 (9.4)	10 (9.0)
Primary language			
English	45 (77.6)	36 (67.9)	81 (73.0)
Spanish	13 (22.4)	17 (32.1)	30 (27.0)
Marital status			
Married	18 (31.0)	16 (30.2)	34 (30.6)
Other (single, divorced, separated, widowed)	40 (70.0)	37 (69.8)	77 (69.4)
Employment status			
Employed	6 (10.3)	1 (1.9)	7 (6.3)
Unemployed (retired, disabled, unemployed)	52 (89.7)	52 (98.1)	104 (93.7)
Insurance type			
Private insurance	4 (6.9)	6 (11.3)	10 (9.0)
Medicare	21 (36.2)	17 (32.1)	38 (34.2)
Medicaid	13 (22.4)	11 (20.8)	24 (21.6)
Uninsured	20 (34.5)	19 (35.8)	39 (35.1)
Body mass index, mean ± SD	35.5 ± 8.0	33.3 ± 8.8	34.5 ± 8.4
Maximum pain level, mean ± SD^b^	7.3 ± 2.0	7.6 ± 2.4	7.4 ± 2.2
Pain location			
Neck	0 (0)	3 (5.7)	3 (2.7)
Upper extremity	3 (5.2)	1 (1.9)	4 (3.6)
Back	19 (32.8)	13 (24.5)	32 (28.8)
Abdomen	1 (1.7)	0 (0)	1 (1.0)
Lower extremity	5 (8.6)	4 (7.5)	9 (8.1)
Multiple areas	30 (51.7)	32 (60.4)	62 (55.9)
Primary outcome, mean ± SD			
Five Times Sit-to-Stand (s)^c^	23.1 ± 13.4^d^	22.1 ± 14.8^d^	22.6 ± 14.0
Secondary outcomes, mean ± SD			
6-Min Walk (ft)^e^	888.1 ± 293.0^f^	1013.2 ± 442.2	948.4 ± 376.0
Borg Perceived Effort^g^	6.2 ± 3.1^h^	5.5 ± 2.8^h^	5.9 ± 3.0
50-ft Speed Walk (s)^c^	19.8 ± 6.8	18.6 ± 5.9	19.3 ± 6.4
SF-12 Physical Component Summary^i^	32.0 ± 7.6	33.7 ± 6.6^j^	32.8 ± 7.2
Patient-Specific Functional Scale^k^	3.3 ± 2.3	3.6 ± 2.4^l^	3.4 ± 2.3
Symbol–Digit Modalities Test^m^	31.7 ± 12.5	30.0 ± 11.0	30.9 ± 11.8

^a^No difference (*P* < 0.05) on any baseline characteristics or measures between two study arms

^b^Maximum pain in 24 h on 11 point numerical rating scale. Higher scores indicate higher levels of pain

^c^Lower Five Times Sit-to-Stand: scores indicate faster completion of test and better physical function

^d^Data missing for three patients in the clinic and one patient in the community

^e^Higher 6-Min Walk: scores indicate ability to walk farther within time frame and better physical function

^f^Data missing for one patient in the community

^g^Modified Borg Perceived Effort: score range is 0–10. Higher scores indicate greater effort to accomplish task

^h^Data missing for one patient in the clinic and two patients in the community

^i^12-Item Short-Form Physical Component Summary: Scores range from 0 to 100. Higher scores indicate better physical performance and capacity

^j^Data missing for two patients in the clinic

^k^Patient-Specific Functional Scale: Scores range from 0 to 10. Higher scores indicate better activity performance

^l^Data missing for two patients in the clinic

^m^Symbol–Digit Modalities Test: Scores range from 0 to 110. Higher scores indicate better cognitive function

At baseline, study subjects in the two arms did not differ significantly on any physical or cognitive measure. The 5XSTS test required a mean of 23 s (SD = 14), and four subjects could not perform this test at baseline. On the 6 MW, subjects in the clinic arm walked somewhat farther with less effort on the Borg Effort test than those in the community arm (Table 1). On other measures, the two study arms differed little. No significant baseline differences appeared between 69 persons with and 42 persons without follow-up measures (Online Appendix B).

In unadjusted analyses among subjects completing 6-month measures, performance on five of seven outcome measures improved significantly (Table 2). On the 5XSTS test, subjects in both arms completed the test faster at 6 months, but only the community arm was also faster at 3 months. Conversely, only the clinic arm subjects improved significantly at 6 months on the 6 MW, Borg Effort test, and SF-12 PCS. The score on the SDMT was significantly increased in both study arms at 6 months.

**Table 2:** Unadjusted Change in Functional Measures in Community and Clinic Arms at 3 and 6 months

Outcomes	Community arm				Clinic arm				All				
Change from baseline	No.	Mean (SD)	*P* value*	Effect size	No.	Mean (SD)	*P* value*	Effect size	No.	Mean (SD)	*P* value*	Effect size	*P* value^†^
5XSTS (s)^‡^													
3 Months	35	−4.53 (6.78)	<0.001	0.67	32	−5.20 (17.53)	0.10	0.30	67	−4.85 (12.97)	0.003	0.37	0.67
6 Months	32	−4.18 (6.45)	0.001	0.65	30	−6.43 (14.11)	0.02	0.46	62	−5.27 (10.82)	<0.001	0.49	0.57
6 MW (ft)^§ǁ^													
6 Months	34	20.0 (291.9)	0.69	0.07	31	193.9 (429.0)	0.02	0.45	65	103.0 (371.3)	0.03	0.28	0.08
Borg Effort^§¶^													
6 Months	34	−0.21 (3.60)	0.74	0.06	29	−1.76 (4.19)	0.03	0.42	63	−0.92 (3.93)	0.07	0.23	0.18
50FtSW (s)^‡^													
3 Months	36	−1.58 (5.55)	0.10	0.28	33	−1.22 (4.29)	0.11	0.28	69	−1.41 (4.95)	0.02	0.28	0.90
6 Months	36	−1.32 (7.16)	0.28	0.18	31	−1.42 (4.85)	0.11	0.29	67	−1.37 (6.15)	0.07	0.22	0.88
SF-12 PCS^§#^													
6 Months	35	2.20 (8.56)	0.14	0.26	30	6.76 (9.99)	0.001	0.68	65	4.31 (9.45)	0.001	0.46	0.06
PSFS^**^													
3 Months	36	1.07 (1.95)	0.002	0.55	32	1.04 (1.66)	0.001	0.63	68	1.05 (1.80)	<0.001	0.58	0.75
6 Months	36	1.64 (2.28)	<0.001	0.72	30	1.65 (1.77)	<0.001	0.93	66	1.64 (2.05)	<0.001	0.80	0.86
SDMT^††^													
3 Months	36	4.19 (6.61)	<0.001	0.63	32	6.84 (10.0)	<0.001	0.68	68	5.44 (8.42)	<0.001	0.65	0.19
6 Months	36	4.61 (8.67)	0.003	0.53	30	7.37 (8.94)	<0.001	0.82	66	5.86 (8.84)	<0.001	0.66	0.10

Abbreviations: *5XSTS* Five Times Sit-to-Stand, *6 MW* 6-Min Walk, *Borg Effor*t Borg Perceived Effort Test, *50FtSW* 50-ft Speed Walk, *SF-12 PCS* 12-Item Short-Form Physical Component Summary, *PSFS* Patient-Specific Functional Scale, *SDMT* Symbol–Digit Modalities Test

*One-sample *t* test was used for examining the changes for clinic and for community, separately

^†^Mann–Whitney *U* test was used for examining the differences between two arms

^‡^Lower scores indicate faster completion of test and better physical function

^§^Measures only assessed at baseline and 6 months

^ǁ^Higher scores indicate ability to walk farther within time frame and better physical function

^¶^Modified score range is 0–10. Higher scores indicate greater effort to accomplish task

^#^12-Item Short-Form Physical Component Summary. Scores range from 0 to 100. Higher scores indicate better physical performance and capacity

^**^Patient-Specific Functional Scale: Scores range from 0 to 10. Higher scores indicate better activity performance

^††^Symbol–Digit Modalities Test: Scores range from 0 to 110. Higher scores indicate better cognitive function

The corresponding effect size for the 5XSTS approached moderate (Cohen’s *d* = 0.49), but was higher for the community arm (Cohen’s *d* = 0.65; Table 2). For the clinic arm at 6 months, the effect size was moderate for the SF-12 PCS (Cohen’s *d* = 0.68) but large for the SDMT (Cohen’s *d* = 0.82) and PSFS (Cohen’s *d* = 0.93). For the SDMT, the effect size was moderate only for the community arm (Cohen’s *d* = 0.53).

**Figure 2:**
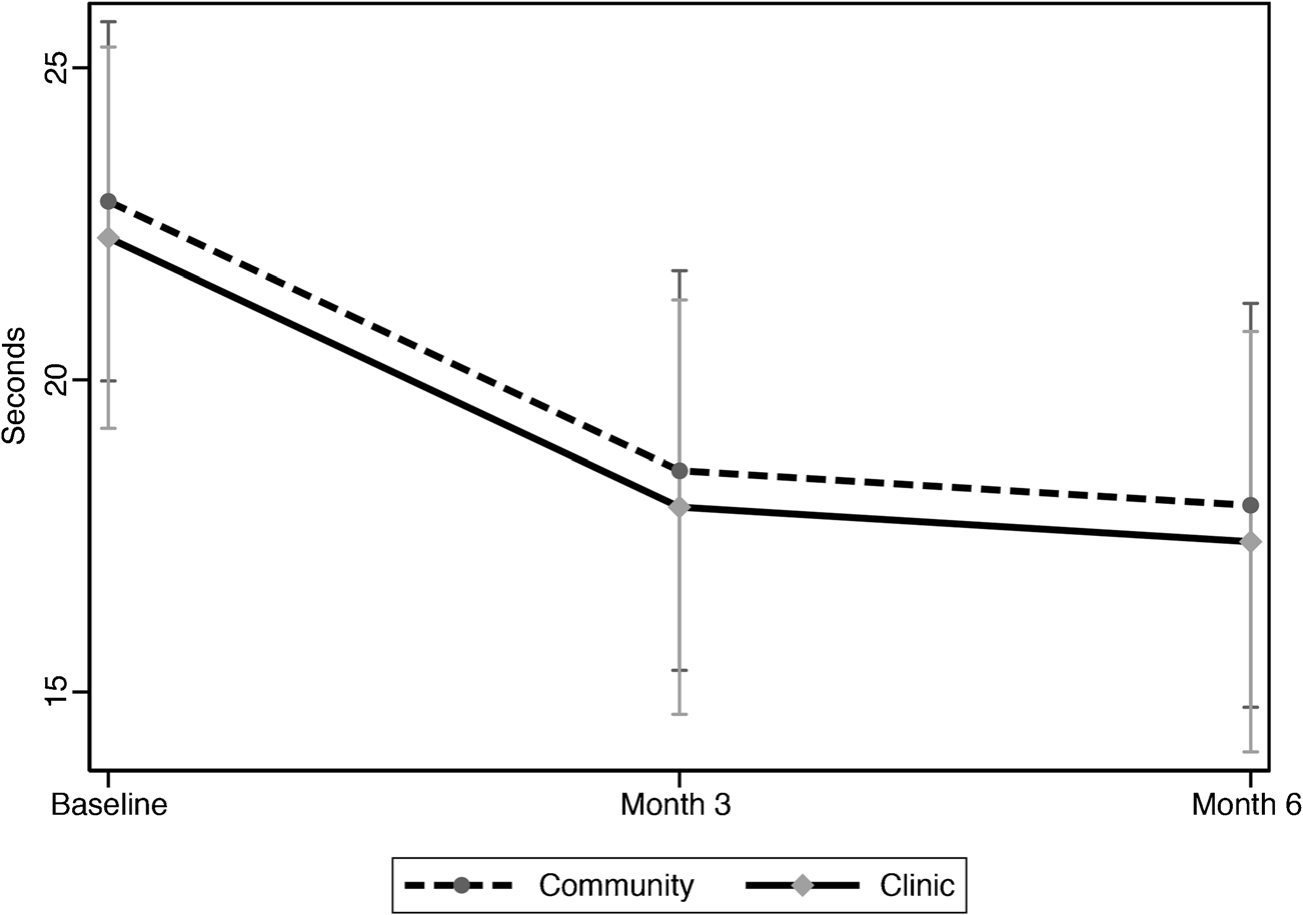
Model-based estimated mean five times sit-to-stand test (5XSTS) performance (s) over time with 95% confidence intervals. Linear mixed-effects model adjusted for time, group, sex, ethnicity, baseline age, baseline body mass index, and baseline maximum pain. Vertical lines indicate 95% confidence intervals.

In adjusted ITT analyses including all 111 randomized subjects (Table 3), performance on the 5XSTS was estimated to average 4–5 s faster at both 3 and 6 months, without and with imputation of missing values (Fig. 2). Only subjects in the clinic walked farther on the 6 MW in all models (*P* ≤ 0.04; Fig. 3.1). The mean score on the Borg Effort test decreased after the 6 MW for both groups at 6 months, but was significant only before imputation (*p* = 0.02; Fig. 3.2). The improvement on the 50FtSW test was not significant (Fig. 3.3). In the clinic arm only, the mean SF-12 PCS improved by an estimated six points in all models (*P* ≤ 0.001; Table 3; Fig. 3.4). In both study arms, the mean PSFS score was estimated 1.6–1.7 points higher at 6 months (both *P* < 0.001; Fig. 3.5), and the mean SDMT score was 5.9–7 points higher at 3 and 6 months (*P* < 0.001; Fig. 3.6).

**Table 3:** Intention-to-Treat Analysis in Linear Mixed-Effects Models With and Without Imputation*

Outcome	Change From baseline score	Without imputation		With imputation	
		Mean (SE)	*P* value	Mean (SE)	P value
5XSTS (s)^†^	3 months	−4.3 (1.3) s	0.003	−3.5 (1.5) s	0.02
	6 months	−4.9 (1.3) s	0.001	−4.0 (1.9) s	0.04
6 MW (ft), clinic^‡§^	6 months	172.4 (61.9) ft	0.02	150.6 (70.8) ft	0.04
Borg Effort^ǁ^	6 months	−1.00 (0.40) points	0.02	−0.98 (0.52) points	0.07
50FtSW (s)^†^	3 months	−1.30 (0.63) s	0.12	−0.94 (0.87) s	0.29
	6 months	−1.20 (0.63) s	0.15	−0.92 (0.86) s	0.28
SF-12 PCS (clinic)^¶#^	6 months	6.2 (1.5) points	<0.001	6.3 (1.8) points	0.001
PSFS^**^	3 months	1.0 (0.2) points	<0.001	0.9 (0.3) points	0.003
	6 months	1.6 (0.2) points	<0.001	1.7 (0.3) points	<0.001
SDMT^††^	3 months	5.6 (1.0) points	<0.001	5.0 (1.3) points	<0.001
	6 months	5.9 (1.0) points	<0.001	7.0 (1.5) points	<0.001

Abbreviations: *5XSTS* Five Times Sit-to-Stand, *6 MW* 6-Min Walk, *Borg Effort* Borg Perceived Effort Test, *50FtSW* 50-ft Speed Walk, *SF-12 PCS* 12-Item Short-Form Physical Component Summary, *PSFS* Patient-Specific Functional Scale, *SDMT* Symbol–Digit Modalities Test

*All linear mixed-effects models adjusted for time, group, sex, ethnicity, baseline age, baseline BMI, and baseline maximum pain unless noted otherwise

^†^Lower scores indicate faster completion of test and better physical function

^‡^Model adjusted for time, group, time × group, sex, ethnicity, baseline age, baseline BMI, and baseline maximum pain. Significant improvement only in clinic arm

^§^Higher scores indicate ability to walk farther within time frame and better physical function

^ǁ^Modified score range is 0–10. Higher scores indicate greater effort to accomplish task

^¶^Model adjusted for time, group, time × group, sex, survey language, baseline age, baseline BMI, and baseline maximum pain. Significant improvement only in clinic arm

^#^12-Item Short-Form Physical Component Summary. Scores range from 0 to 100. Higher scores indicate better physical performance and capacity

^**^Patient-Specific Functional Scale: Scores range from 0 to 10. Higher scores indicate better activity performance

^††^Symbol–Digit Modalities Test: Scores range from 0 to 110. Higher scores indicate better cognitive function

**Figure 3:**
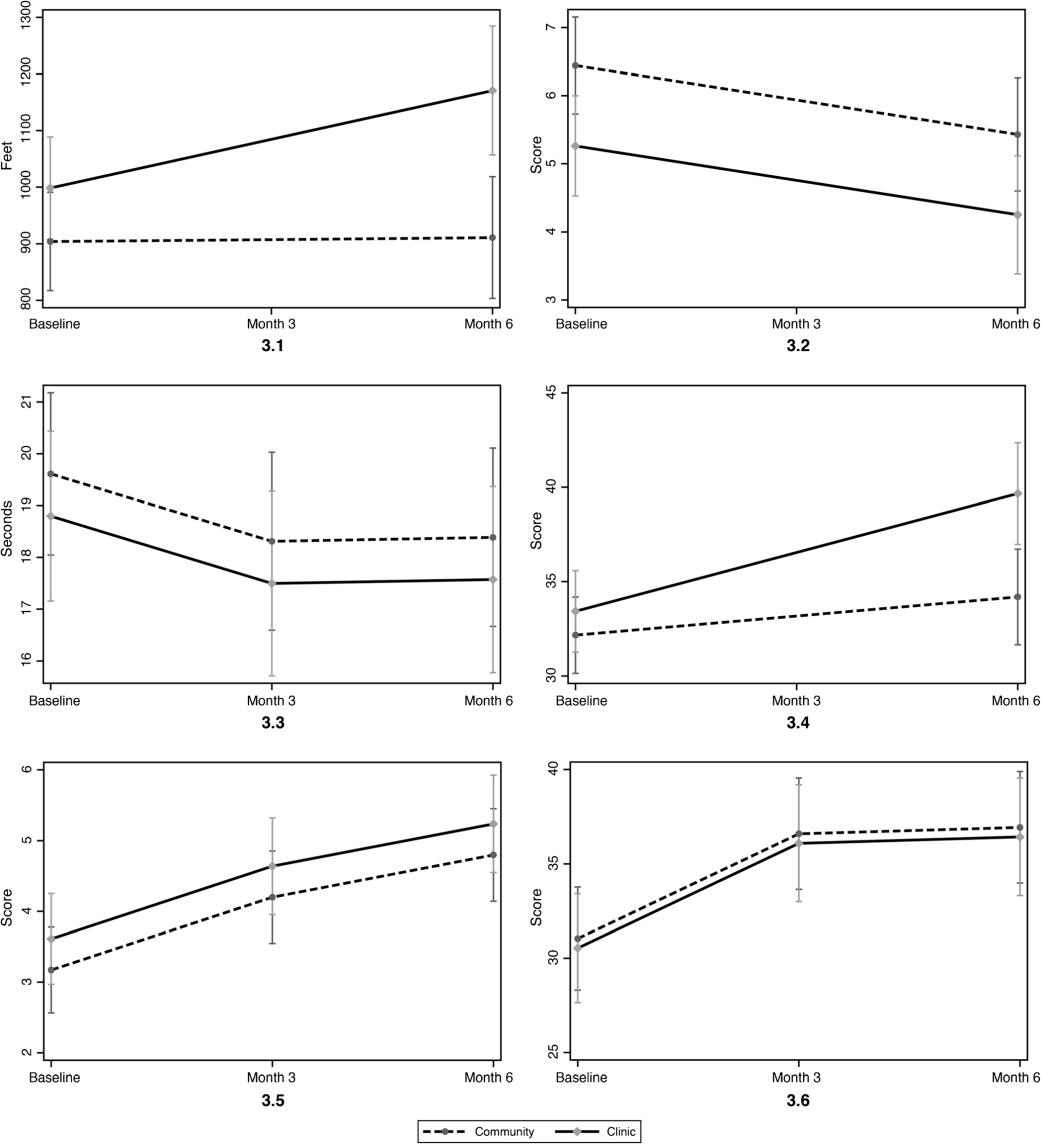
Model-based estimated mean secondary outcomes over time. Linear mixed-effects models adjusted for time, group, sex, ethnicity, baseline age, baseline BMI, and baseline maximum pain for 50-ft speed walk, Borg Perceived Effort, Patient-Specific Functional Scale, and Symbol–Digit Modalities Test. Model adjusted for time, group, time × group, sex, ethnicity, baseline age, baseline body mass index, and baseline maximum pain for 6-min walk. Model adjusted for time, group, time × group, sex, survey language, baseline age, baseline BMI, and baseline maximum pain for 12-Item Short-Form Physical Component Summary (SF-12 PCS). Vertical lines indicate 95% confidence intervals. Figure 3.1 6-Min walk. Figure 3.2 Borg Perceived Effort. Figure 3.3 50-Ft speed walk. Figure 3.4 12-Item Short-Form Physical Component Summary. Figure 3.5 Patient-Specific Functional Scale. Figure 3.6 Symbol–Digit Modalities Test.

Six subjects in the community arm and two in the clinic arm experienced adverse events. In the community arm, these included 1) a fractured leg from a fall in a patient who tripped when entering her house; 2) a fall at a holiday gathering at home; 3) a fall after losing balance when standing from a chair; 4) a fall at a store after losing balance; 5) amputation due to a diabetic foot infection; and 6) gout attack requiring hospitalization. In the clinic arm, events included a recurrent wrist injury requiring a brace and a fall at home prior to attending any meetings. Only one fall resulted in significant injury.

## DISCUSSION

Low-income community stakeholders with chronic pain have limited resources for improving their functional status.^5^ This trial examined two approaches for delivering a chronic pain self-management training program that was developed de novo to help address local community stakeholders’ needs. Our primary outcome was the 5XSTS, which assesses lower limb function, coordination, and balance,^6^^,^ 11 ^,^ 12 and is predictive of falls in the elderly^23^ and of future disability.^24^ Our study subjects performed poorly on the 5XSTS test at baseline, requiring 23 s versus only 13 s in older women with fibromyalgia.^25^ However, in an ITT analysis, subjects in both study arms improved significantly on 5XSTS tests, by 4–5 s at 3 and 6 months, which exceeds a minimum clinically important difference (MCID) of 2.5 s on the 5XSTS test.^26^^,^ 27 Subjects in both arms also demonstrated significant improvement at both time points on the PSFS, which is a reliable and valid measure of functional change for musculoskeletal disorders.^28^ The 1.6-point increase in the PSFS score falls between a small (>0.8) and medium (>3.2) MCID value.^28^

The SDMT evaluates psychomotor processing speed, which is impaired in chronic pain and fibromyalgia.^29^^–^ 31 All study subjects performed poorly on the SDMT at baseline, with a mean score of 30.9, compared with 36.7 on normative data for persons aged 51–65 with <12 years of education.^29^ In both study arms, the mean SDMT score increased by an estimated six points at 6 months, which is both statistically and clinically significant.^32^

Despite a greater number of educational sessions for the community arm than the clinic arm, only clinic arm subjects had statistically and clinically significant improvement on the 6 MW test and SF-12 PCS in ITT analyses. The clinic arm subjects averaged 172 ft or 52.4 m farther, exceeding the MCID value of 14.0–30.5 m reported in a review.^33^ The mean 12-PCS score increased by an estimated 6.2 points, which is above the range of 3.2–6.1 for an MCID.^34^ These results add to evidence supporting the role of the CHW as a health educator for persons with chronic disease, especially in communities with limited resources.^35^ Compared with chronic pain self-management support from nurse practitioners,^36^ CHWs may be more feasible for practices serving low-income communities.

We did not offer a control arm, because clinic directors wanted to help their disabled patients. The moderate to large effect sizes for most of our outcome measures stand in marked contrast to the lack of significant effects for other chronic pain self-management interventions.^37^^,^ 38 In a systematic review, self-management programs for osteoarthritis had no or only small benefits.^39^ The apparent success of our program may also reflect a focus on addressing the unmet needs of community stakeholders from low-resource communities. We integrated elements of a functional restoration intervention to improve pain-coping skills, increase physical functioning, and promote a return to an active, engaged lifestyle, but did not offer formal cognitive-behavioral therapy as described by Gatchel and Mayer.^2^

Although few adverse events were reported in a review of self-management for osteoarthritis,^39^ five of our study subjects experienced falls. These falls occurred during usual activities, so it is unclear whether patients were more active than usual because of the program. We instructed the patients to perform only those activities that they felt safe in doing. Study subjects did improve on the 5XSTS test, which has been associated with reduced risk of falls in the elderly.^23^ Nevertheless, future research should be attentive to fall risk and should offer support and training to reduce this risk.

Other limitations of this trial included challenges in recruiting and retaining subjects. Half of potentially eligible subjects could not be reached by telephone, and about one-third of subjects dropped out after baseline assessment. In addition, two-thirds of participants needed to make up missed sessions. In a study of physical therapy for neck or back pain, only 60% of subjects adhered to prescribed visits.^40^ In fact, a systematic review has called for specific interventions to address non-adherence to exercise for chronic pain.^41^ Patients may prioritize treatment with pain medications over non-pharmacologic interventions. In our survey of a representative sample of Hispanics without chronic pain from five states, those who reported having greater knowledge about chronic pain were more likely to endorse relying on pain medications for management.^42^ It is incumbent on primary care physicians to help their patients understand that living with chronic pain requires active self-management on a daily basis.

Other limitations include the lack of long-term follow-up and potential lack of generalizability of a single-institution study. In view of the critical need to reduce reliance on opioids, future studies should evaluate whether this self-management program is helpful for tapering and even eliminating opioids.^43^ Additionally, the difference in the level of expertise between the individuals delivering our program in each of the two study arms could be seen as a limitation, but we intentionally designed the trial to examine outcomes from these two approaches. The team members were not blinded to study arm, but outcome measurements were conducted by members who had no role in that study arm. Lastly, subjects often missed sessions, so we accommodated with make-up sessions; however, the impact of this approach could not be assessed.

The success of this program may lie in providing subjects with training on multiple self-management methods, since no single non-pharmacologic intervention for chronic pain has shown a consistently large effect.^44^ The program resulted in clinically important changes in multiple objective measures, in contrast to other chronic pain and arthritis self-management studies that have examined self-reported measures.^45^ Our easily replicable self-management program may represent a promising resource to help low-income patients with chronic pain adopt a more proactive lifestyle to manage this debilitating disease.

## Electronic supplementary material

ESM 1 (DOCX 22 kb)

## References

[1] Dowell D, Haegerich TM, Chou R (2016). CDC guideline for prescribing opioids for chronic pain—United States, 2016. JAMA..

[2] Gatchel RJ, Mayer TG (2008). Evidence informed management of chronic low back pain with functional restoration. Spine J..

[3] Wallerstein NB, Duran B (2006). Using community-based participatory research to address health disparities. Health Promot Pract..

[4] Koh HK, Oppenheimer SC, Massin-Short SB, Emmons KM, Geller AC, Viswanath K (2010). Translating research evidence into practice to reduce health disparities: a social determinants approach. Am J Public Health..

[5] Valerio MA, Rodriguez N, Winkler P (2016). Comparing two sampling methods to engage hard-to-reach communities in research priority setting. BMC Med Res Methodol..

[6] Jones SE, Kon SS, Canavan JL (2013). The five-repetition sit-to-stand test as a functional outcome measure in COPD. Thorax..

[7] Butler DS, Moseley GL (2013). Explain Pain.

[8] The Progressive Goal Attainment Program (PGAP®). An Evidence-Based Treatment Program for Reducing Disability Associated with Pain, Depression, Cancer and other Chronic Health Conditions. Available at: https://www.pgapworks.com/en/whatisthepgap/index.php. Accessed 15 Nov 2017.

[9] **Moore P, Cole F.** The Pain Toolkit. Available at: http://www.paintoolkit.org/. Accessed 15 Nov 2017.

[10] Community Health Association of Mountain/Plains States. Patient Education Handouts. Available at: http://www.champsonline.org/ToolsProducts/ClinicalResources/PatientEdTools/PatientEdHandouts.html. Accessed 15 Nov 2017.

[11] Smeets RJ, Hijdra HJ, Kester AD, Hitters MW, Knottnerus JA (2006). The usability of six physical performance tasks in a rehabilitation population with chronic low back pain. Clin Rehabil..

[12] Bohannon RW (2011). Test-retest reliability of the five-repetition sit-to-stand test: a systematic review of the literature involving adults. J Strength Cond Res..

[13] Steffen TM, Hacker TA, Mollinger L (2002). Age- and gender-related test performance in community-dwelling elderly people: Six-Minute Walk Test, Berg Balance Scale, Timed Up & Go Test, and gait speeds. Phys Ther.

[14] Borg GA (1982). Psychophysical bases of perceived exertion. Med Sci Sports Exerc..

[15] Ware JE, Kosinski M, Keller SD (1995). SF-12: how to Score the SF-12 Physical and Mental Health Summary Scales.

[16] Abbott JH, Schmitt J (2014). Minimum important differences for the patient-specific functional scale, 4 region-specific outcome measures, and the numeric pain rating scale. J Orthop Sports Phys Ther..

[17] Smith A (1982). Symbol digit modalities test (SDMT) manual (revised).

[18] **Van Schependom J, D’hooghe MB, Cleynhens K, et al.** The Symbol Digit Modalities Test as sentinel test for cognitive impairment in multiple sclerosis. Eur J Neurol. 2014;21(9):1219–25, e71–2. 10.1111/ene.12463 24850580

[19] **Turner BJ, Arimendez SV, Liang Y, Simmonds M, Pugh MJ**. Functional outcomes of peer support for veterans on long-term opioids for chronic pain. J Chronic Dis Manag. In press.

[20] Cohen J (1988). Statistical Power Analysis for the Behavioral Sciences.

[21] Raghunathan TE, Lepkowksi JM, Van Hoewyk J, Solenbeger P (2001). A multivariate technique for multiply imputing missing values using a sequence of regression models. Surv Methodol..

[22] Rubin DB (1987). Multiple Imputation for Nonresponse in Surveys.

[23] Buatois S, Miljkovic D, Manckoundia P (2008). Five times sit to stand test is a predictor of recurrent falls in healthy community-living subjects aged 65 and older. J Am Geriatr Soc..

[24] Makizako H, Shimada H, Doi T (2017). Predictive cutoff values of the five-times sit-to-stand test and the timed "Up & Go" test for disability incidence in older people dwelling in the community. Phys Ther..

[25] Dailey DL, Frey Law LA, Vance CG (2016). Perceived function and physical performance are associated with pain and fatigue in women with fibromyalgia. Arthritis Res Ther..

[26] Meretta BM, Whitney SL, Marchetti GF, Sparto PJ, Muirhead RJ (2006). The five times sit to stand test: responsiveness to change and concurrent validity in adults undergoing vestibular rehabilitation. J Vestib Res..

[27] Goldberg A, Chavis M, Watkins J, Wilson T (2012). The five-times-sit-to-stand test: validity, reliability and detectable change in older females. Aging Clin Exp Res..

[28] Horn KK, Jennings S, Richardson G, Vliet DV, Hefford C, Abbott JH (2012). The patient-specific functional scale: psychometrics, clinimetrics, and application as a clinical outcome measure. J Orthop Sports Phys Ther..

[29] González HM, Whitfield KE, West BT, Williams DR, Lichtenberg PA, Jackson JS (2007). Modified-Symbol Digit Modalities Test for African-Americans, Caribbean black Americans, and non-Latino whites: nationally representative normative data from the National Survey of American Life. Arch Clin Neuropsychol..

[30] Rathbone M, Parkinson W, Rehman Y, Jiang S, Bhandari M, Kumbhare D (2016). Magnitude and variability of effect sizes for the associations between chronic pain and cognitive test performances: a meta-analysis. Br J Pain..

[31] Cherry BJ, Zettel-Watson L, Shimizu R, Roberson I, Rutledge DN, Jones CJ (2014). Cognitive performance in women aged 50 years and older with and without fibromyalgia. J Gerontol B Psychol Sci Soc Sci..

[32] Sandroff BM, Klaren RE, Pilutti LA, Dlugonski D, Benedict RH, Motl RW (2014). Randomized controlled trial of physical activity, cognition, and walking in multiple sclerosis. J Neurol..

[33] Bohannon RW, Crouch R (2017). Minimal clinically important difference for change in 6-minute walk test distance of adults with pathology: a systematic review. J Eval Clin Pract.

[34] Parker SL, Adogwa O, Mendenhall SK (2012). Determination of minimum clinically important difference (MCID) in pain, disability, and quality of life after revision fusion for symptomatic pseudoarthrosis. Spine J..

[35] Shah M, Kaselitz E, Heisler M (2013). The role of community health workers in diabetes: update on current literature. Curr Diab Rep..

[36] Broderick JE, Keefe FJ, Bruckenthal P (2014). Nurse practitioners can effectively deliver pain coping skills training to osteoarthritis patients with chronic pain: a randomized, controlled trial. Pain..

[37] Ersek M, Turner JA, Cain KC, Kemp CA (2008). Results of a randomized controlled trial to examine the efficacy of a chronic pain self-management group for older adults [ISRCTN11899548]. Pain..

[38] Morone NE, Greco CM, Moore CG (2016). A mind-body program for older adults with chronic low back pain: a randomized clinical trial. JAMA Intern Med..

[39] Kroon FP, van der Burg LR, Buchbinder R, Osborne RH, Johnston RV, Pitt V (2014). Self-management education programmes for osteoarthritis. Cochrane Database Syst Rev..

[40] Medina-Mirapeix F, Escolar-Reina P, Gascón-Cánovas JJ, Montilla-Herrador J, Jimeno-Serrano FJ, Collins SM (2009). Predictive factors of adherence to frequency and duration components in home exercise programs for neck and low back pain: an observational study. BMC Musculoskelet Disord..

[41] Jordan JL, Holden MA, Mason EE, Foster NE (2010). Interventions to improve adherence to exercise for chronic musculoskeletal pain in adults. Cochrane Database Syst Rev..

[42] Turner BJ, Liang Y, Rodriguez N (2017). Gaps in the public’s knowledge about chronic pain: representative sample of Hispanic residents from 5 states. J Pain..

[43] Westanmo A, Marshall P, Jones E, Burns K, Krebs EE (2015). Opioid dose reduction in a VA Health Care System—implementation of a primary care population-level initiative. Pain Med..

[44] Geneen LJ, Moore RA, Clarke C, Martin D, Colvin LA, Smith BH (2017). Physical activity and exercise for chronic pain in adults: an overview of Cochrane Reviews. Cochrane Database of Syst Rev..

[45] Bair MJ, Ang D, Wu J (2015). Evaluation of Stepped Care for Chronic Pain (ESCAPE) in Veterans of the Iraq and Afghanistan Conflicts: a randomized clinical trial. JAMA Intern Med..

