# Supplementary material for: Randomized Trial of Chronic Pain Self-Management Program in the Community or Clinic for Low-Income Primary Care Patients
Source: J Gen Intern Med. 2018 Jan 3;33(5):668–77. doi: 10.1007/s11606-017-4244-2 (PMC5910333; doi:10.1007/s11606-017-4244-2)
Supplement: Supplementary file 1 — (DOCX 22 kb) [file 11606_2017_4244_MOESM1_ESM.docx]

| **Appendix A. Living Better Beyond Pain Curriculum** | | | |
| --- | --- | --- | --- |
| **Session #** | **Lecture Session** | **Content** | **Community Speaker** |
| **1** | **Understanding Chronic Pain and Goal Setting** | - Successful and unsuccessful pain strategies- testimonials from patients - Personal goals for daily activities with pain - Pain: chronic versus acute pain - Mind-body pain connection - Opioids and non-drug therapies and relative benefits for pain - Non-pharmacologic ways to control pain - Proper posture and walking - Chronic pain management for a fuller life | General Internist |
| **2** | **Pain and Physical Activity** | - Core muscles and pain management - Incorrect posture and pain - Physical activity and its benefits for pain management - Recommended physical activities - Proper form for walking, stretching, and strengthening - Safety measures for walking, stretching and strengthening | Kinesiology Professor |
| **3** | **Meditation and Mindfulness** | - Overview of the role of the mind in pain management - Unhelpful thinking habits and ways to overcome them - Mindfulness and impact on managing pain - Mindfulness exercises - Stress and relaxation - Ways to relax and focus on positive thoughts - Mindfulness techniques to support pain self-management | Psychology Assistant Professor |
| **4** | **Massage Techniques** | - Types of massage - Benefits of massage therapy for pain management - Trigger points and different ways to massage, demonstrations - Different ways to practice massage on your own - Tools for massage at home - How to get down and up off of the floor safely | Certified Masseuse |
| **5** | **Nutrition** | - Nutrition and pain management connection - Examples of a “healthy plate” - Foods that are part of a healthy lifestyle - Simple, healthy approaches to eat - Moderation and portion control - Examples of reducing portion size  ​ - Food substitutions and examples | Dietician |
| **6** | **Prevention and Management of Set Backs*** | - Setbacks and pain management - Unhealthy ways to handle set backs - Ways to overcome set backs - Creating a plan for set backs | Physical Therapy Professor |
| **7** | **Sleep Hygiene** | - Sleep hygiene and how it affects pain - Unhealthy sleeping habits ​ - Tips for sleeping better - Daytime and nighttime routine for better sleep - Negative effects of sleeping pills - Cognitive behavioral therapy and sleep | General Internist |
| **8** | **Health Literacy*** | - Health literacy and finding support resources - How to access data in the Internet - Examples of resources for chronic pain - Caution about recommendations in the Internet - Work with health care provider to review recommendations | Librarian |
| **9** | **Looking Back** | - Program goals and objectives - Progress during the program - Practicing ways to control pain - Lessons learned to help overcome setbacks - Personal and community resources to help them with pain improvement journey - Daily personalized plans for patients | General Internist |

*These sessions were only delivered to the community arm

| **Appendix B: Comparison of baseline characteristics for subjects with only baseline measures versus subjects having at least one post baseline measures** | | | | |
| --- | --- | --- | --- | --- |
| Characteristics | All  N=111 | Participated in Baseline Only  N = 42 | Participated in 3 / 6 Month Measures  N = 69 | P Value^*^ |
| **Study Arm** |  |  |  |  |
| Community | 58 (52.3) | 22 (52.4) | 36 (52.2) | 1^†^ |
| Clinic | 53 (47.8) | 20 (47.6) | 33 (47.8) |  |
| **Age**, mean±SD (years) | 56.5±9.0 | 54.7±10.5 | 57.7±7.9 | 0.19^*^ |
| **Women** | 61 (55.0) | 22 (52.4) | 39 (56.5) | 0.82^†^ |
| **Race/Ethnicity,** n(%) |  |  |  |  |
| Hispanic | 87 (78.4) | 36 (85.7) | 51 (73.9) | 0.54^†^ |
| Non-Hispanic White | 14 (12.6) | 4 (9.5) | 10 (14.5) |  |
| Non-Hispanic Black | 10 (9.0) | 2 (4.8) | 8 (11.6) |  |
| **Primary Language,** n(%) |  |  |  |  |
| English | 81 (73.0) | 30 (71.4) | 51 (73.9) | 0.95^†^ |
| Spanish | 30 (27.0) | 12 (28.6) | 18 (26.1) |  |
| **Marital Status,** n(%) |  |  |  |  |
| Married | 34 (30.6) | 12 (28.6) | 21 (30.4) | 1^†^ |
| Other (single, divorced, separated, widowed) | 77 (69.4) | 30 (71.4) | 48 (69.6) |  |
| **Employment Status** |  |  |  |  |
| Employed | 7 (6.3) | 2 (4.8) | 5 (7.2) | 0.91^†^ |
| Unemployed (retired, disabled, unemployed) | 104 (93.7) | 40 (95.2) | 64 (92.8) |  |
| **Insurance Type** |  |  |  |  |
| Insured | 10 (9.0) | 6 (14.3) | 4 (5.8) | 0.35^†^ |
| Medicare | 38 (34.2) | 10 (23.8) | 28 (40.6) |  |
| Medicaid | 24 (21.6) | 9 (21.4) | 15 (21.7) |  |
| Uninsured (self-pay/carelink) | 39 (35.1) | 17 (40.5) | 22 (31.9) |  |
| **Body Mass Index**, mean±SD | 34.5±8.4 | 35.1±9.4 | 34.1±7.8 | 0.97^*^ |
| **Maximum pain level,** mean±SD^‡^ | 7.40±2.2 | 7.40±1.9 | 7.50±2.4 | 0.99^*^ |
| **Pain Location,** n(%) |  |  |  |  |
| Neck | 3 (2.7) | 2 (4.8) | 1 (1.4) | 0.85^†^ |
| Upper extremity | 4 (3.6) | 2 (4.8) | 2 (2.9) |  |
| Back | 32 (28.8) | 13 (31.0) | 19 (27.5) |  |
| Abdomen | 1 (1.0) | 1 (2.4) | 0 (0) |  |
| Lower extremity | 9 (8.1) | 1 (2.4) | 8 (11.6) |  |
| Multiple areas | 62 (55.9) | 23 (54.8) | 39 (56.5) |  |
| **Primary Outcome,** **mean±SD** |  |  |  |  |
| 5 times sit-to-stand (seconds)^§^ | 22.6±14.0 | 21.2±14.9^ǁ^ | 23.5±13.4^ǁ^ | 0.12^*^ |
| **Secondary Outcomes, mean±SD** |  |  |  |  |
| 6-minute distance walk (feet)^¶^ | 948.4±376.0 | 956.8±405.5 | 943.2±359.5^#^ | 0.95^*^ |
| Borg Perceived Effort^**^ | 5.9±3.0 | 5.8±3.1^††^ | 5.9±2.9^††^ | 0.85^*^ |
| 50-foot speed walk (seconds)^§^ | 19.3±6.4 | 18.9±6.8 | 19.5±6.2 | 0.49^*^ |
| 12-Item Physical Component Summary^‡‡^ | 32.8±7.2 | 33.0±7.2^a^ | 32.7±7.2^a^ | 0.76^*^ |
| Patient Specific Functional Scale^b^ | 3.4±2.3 | 3.4±2.5^c^ | 3.4±2.3 | 0.93^*^ |
| Symbol Digit Modalities Test^d^ | 30.9±11.8 | 31.2±11.4^e^ | 30.7±12.1 | 0.82^*^ |

^*^ Mann-Whitney U test.

^†^ Chi-squared test.

^‡^ Maximum pain in 24 hours on 11 point numerical rating scale. Higher scores indicate higher levels of pain.

^§^ Lower scores indicate faster completion of test and better physical function.

^ǁ^ Data missing for two patients in baseline group and two patients in post baseline group

^¶^ Higher scores indicate ability to walk farther within timeframe and better physical function.

^#^ Data missing for one patient in post baseline group

^**^ Modified score range is 0-10. Higher scores indicate greater effort to accomplish task.

^††^ Data missing for one patient in the baseline group and two patients in the post baseline group

^‡‡^ 12-Item Short Form Physical Component Summary. Scores range from 0-100. Higher scores indicate better physical performance and capacity.

^a^ Data missing for one patient in baseline group and one patient in post baseline group

^b^ Patient Specific Functional Scale: Scores range from 0-10. Higher scores indicate better activity performance.

^c^ Data missing for two patients in baseline group

^d^ Symbol Digit Modalities Test: Scores range from 0-110. Higher scores indicate better cognitive function.

^e^ Data missing for one patient in baseline group
